# Influence of the number of images on threshold image contrast measurements with a phantom with gold disks in digital mammography

**DOI:** 10.1007/s12194-025-00958-3

**Published:** 2025-09-02

**Authors:** Michał Biegała, Agata Batolik

**Affiliations:** 1 Faculty of Medicine, Department of Medical Imaging Technology, Medical University of Lodz, Narutowicza 60, 90-136 Lodz, Poland; 2 Department of Medical Physics, Copernicus Memorial Hospital in Lodz Comprehensive Cancer Center and Traumatology, Lodz, Poland; 3 Department of Radiotherapy, Copernicus Memorial Hospital in Lodz Comprehensive Cancer Center and Traumatology, Lodz, Poland

**Keywords:** Mammography, Quality control, CDMAM phantom, Threshold image contrast

## Abstract

Image quality, in addition to radiation dose, is the most important physical parameter in digital mammography. Image quality should be periodically monitored using the CDMAM phantom. The aim of this study is to investigate the effect of the number of analyzed images on the result of threshold image contrast measurements using the CDMAM phantom in different versions. The images obtained using two versions of the CDMAM phantom, i.e., 3.4 and 4.0, were analyzed. The image analysis was performed and repeated 10 times for 2, 4, 6, 8, 12, 16, 24, and 32 images from a pool of 43 images, separately for each phantom. For the CDMAM 3.4 phantom, a statistical difference was demonstrated between the following groups: S2 vs S6 (*p* < 0.006), S6 vs S16 (*p* < 0.001), S6 vs S24 (*p* < 0.002), S6 vs S32 (*p* < 0.021), S8 vs S16 (*p* < 0.019), S8 vs S24 (*p* < 0.048). For the CDMAM 4.0 phantom, a statistically significant difference was demonstrated between all groups and the N2 group (*p* < 0.000). For the CDMAM 3.4 phantom, the most favorable number of images required for analysis cannot be clearly determined. For the CDMAM 4.0 phantom, it is recommended to perform 24 images for analysis. Particular attention should be paid when determining the threshold image contrast for a disk diameter of 0.1 mm, as this parameter is used during exposure automation control.

## Introduction

Image quality analysis is, next to the ionizing radiation dose, the most important physical parameter that should be periodically verified in mammography [1–3]. Image quality in mammography is defined by the threshold image contrast, which has a significant impact on the diagnostic quality of examinations performed in women [4, 5]. Threshold image contrast refers to the lowest contrast level at which a given object (disk) is still detectable by the system under defined imaging conditions. It is derived using software analysis of phantom images and serves as a quantitative indicator of image quality in mammography. It is very important to assess image quality correctly using precisely made phantoms or measuring tools and to apply appropriate procedures for assessing the obtained results [6–8]. One of the phantoms used to assess image quality is the CDMAM phantom from Artinis with appropriate computer software allowing for a reliable assessment of image quality [9]. This phantom is recommended by the European Reference Organization for Quality Assured Breast Screening and Diagnostic Services (EUREF) for assessing image quality in mammography [10]. Image quality assessment performed using this phantom consists in taking several images of the CDMAM phantom with specific exposure parameters and then analyzing them using appropriate software that determines the threshold image contrast. The value of the threshold image contrast determined by the software depends, among other things, on the number of images analyzed by the software, which is an important parameter in determining the threshold image contrast. The aim of this work is to investigate the influence of the number of analyzed images on the result of the threshold image contrast measurements using the CDMAM phantom in different versions [11]. This issue is of particular relevance in clinical quality control routines, where time constraints and varying recommendations from phantom manufacturers often lead to differences in the number of images analyzed. Despite the use of CDMAM phantoms being standardized under EUREF guidelines, there remains a lack of consensus in practice, especially regarding minimum image count. Clarifying the impact of image count on contrast measurements is therefore essential for harmonizing QA practices and ensuring reliable results without unnecessarily prolonging testing procedures. This study is a continuation of our previous work [11], which compared absolute contrast values obtained using CDMAM 3.4 and 4.0 phantoms on different types of mammographs. In the present study, we focused specifically on the effect of image count on measurement stability and precision, with detailed statistical analysis across multiple group sizes. Unlike the previous comparison of phantom versions, this work provides evidence-based guidance on the optimal number of images for quality control procedures using each phantom version.

## Material and methods

The images obtained using two versions of CDMAM phantoms, i.e., version 3.4 and 4.0, were analyzed. The CDMAM 3.4 phantom is made of an aluminum plate and 4 PMMA (poly(methyl methacrylate)) plates 10.0 ± 0.1 mm thick to maintain the scattering conditions. The aluminum plate of the CDMAM 3.4 phantom contains 205 cells. In the center of each cell and in a randomly selected corner of it, there is a gold disk of a specified diameter and thickness. In the CDMAM 3.4 phantom, the objects (gold disks) have a diameter from 0.1 to 2.3 mm and a thickness from 0.05 to 1.6 μm, which in the anode/Mo/Mo filter conditions gives a contrast range of 1–25% [12]. The CDMAM 4.0 phantom is also made of an aluminum plate and 4 plates with a thickness of 10.0 ± 0.1 mm made of PMMA. However, the aluminum plate of the CDMAM 4.0 phantom contains 336 cells in which gold discs are placed in a similar way as in the phantom version 3.4. The gold discs in the newer version of the phantom have a diameter of 0.06 to 2.0 mm and a thickness of 0.03 to 2.0 μm, which gives a contrast range of 0.5–30% in the standard mammographic exposure conditions [13]. The total thickness of the phantom corresponds to 5 cm of the breast after compression. The phantoms differ from each other not only in the number of cells with gold discs but also in their geometric arrangement on the plate. Each phantom allows determining the threshold image contrast of discs with a diameter of 0.1 mm, 0.25 mm, 0.5 mm, 1 mm, and 2 mm. Disks of the same diameter allow for comparison of contrast values using the CDMAM 3.4 and CDMAM 4.0 phantoms [9, 14–16], in accordance with EUREF recommendations.

In this work, images from two versions of the CDMAM phantom were analyzed. To eliminate software-related errors, images were read using software from one manufacturer. For this purpose, the CDMAM Analysis v2.3.0 software from the National Coordinating Centre for the Physics of Mammography, Guildford, UK (NCCPM) was used, which allows for analysis of images taken with the CDMAM 3.4 and CDMAM 4.0 phantoms, using cdcom 1.6 and cdcom 4 files.

For each version of the CDMAM phantom, 43 images were taken on a Selenia Dimensions mammography from Lorad with an a-Se (DR) detector. Images were taken with exposure parameters corresponding to the breast thickness after compression of 50 mm (according to the phantom manufacturer's recommendations), i.e., 32 kV, 140 mAs and anode/filter combination W/Rh.

All images were acquired under controlled and repeatable conditions. Environmental parameters such as room temperature and equipment warm-up time were kept constant throughout the image acquisition process. The same mammography system and exposure parameters were used for all images. A single experienced operator conducted the acquisitions to ensure consistency and reduce operator-dependent variability. The CDMAM phantom was carefully positioned using alignment markers on the mammographic table, and its position was verified before each exposure. To minimize the influence of detector-specific artifacts and ensure sampling independence, the phantom was shifted in a standardized clockwise pattern by 5 mm between subsequent exposures, as described below.

**Fig. 1:**
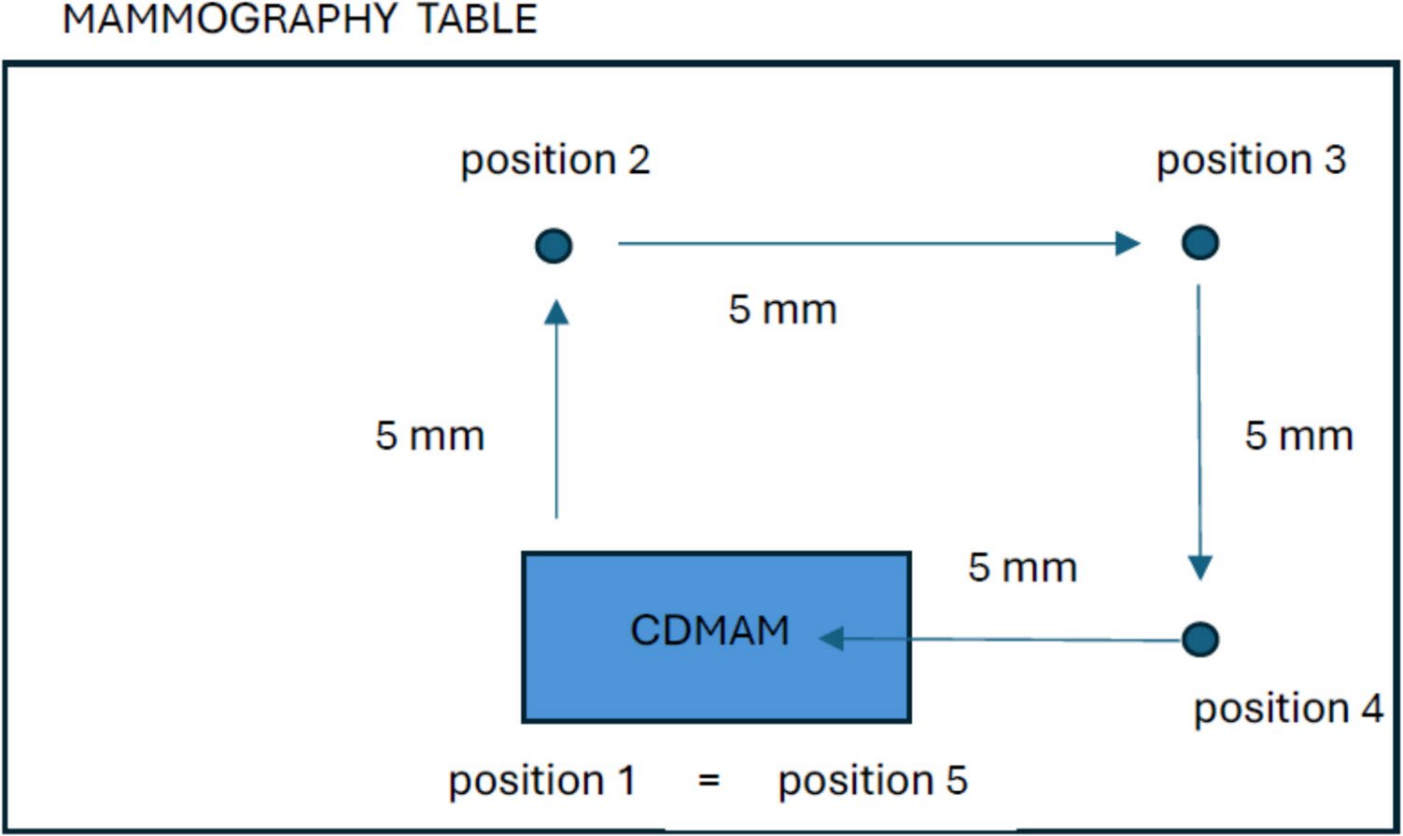
The movement of the CDMAM phantom on the mammographic table

Different numbers of recorded images of CDMAM phantoms from a pool of 43 images, selected and randomly sampled using a random number generator, were analyzed. Any repetitions of sequences of numbers in each sample were verified and eliminated on an ongoing basis. Image analysis was repeated 10 times for each group size (2, 4, 6, 8, 12, 16, 24, and 32 images) using randomly selected subsets from the pool of 43 images for each phantom type. All analyzed images were in the “for processing” mode – unprocessed images. Before each exposure, the phantom was moved to eliminate detector-related artifacts from the image. The phantom was moved clockwise by 5 mm, i.e., first position – exactly in the center of the detector, second position – shift 5 mm up, third position – shift 5 mm to the right, fourth position—shift 5 mm down, fifth position—shift 5 mm to the left. The movement of the CDMAM phantom on the mammographic table is shown in Fig. 1.

Statistical analysis of the results obtained in this work was performed using Statistica 13.3 software. The statistical tools used to compare groups of results were the Tukey’s HSD (Honestly Significant Difference) test and the Least Significant Difference (NIR) test, both belonging to the POST HOC group of multiple comparison procedures. Tukey’s HSD test was chosen for its conservative control of Type I error when comparing multiple groups. The NIR test, being more liberal, was applied in parallel to detect additional possible differences. Where results diverged, we prioritized the findings from the Tukey test, but also reported NIR results to reflect the sensitivity of the analysis to smaller effects.

The level of significance was assumed to be *α* = 0.05; therefore, *p* < 0.05 confirms statistical difference between the tested groups.

## Results

Table 1 presents the average threshold image contrast values obtained during the analysis of different numbers of images of the CDMAM 3.4 phantom. The values are given with ± 2 standard errors (SE). The highest threshold image contrast was observed for a gold disk diameter of 0.1 mm with 8 images analyzed (0.772 ± 0.093), while the lowest value occurred for 16 images (0.735 ± 0.057), resulting in a 5.12% difference. A clear trend of decreasing standard error with an increasing number of images is visible; for example, the SE for 0.10 mm disks decreased from ± 0.223 (2 images) to ± 0.037 (32 images), improving measurement precision.

**Table 1:** Analysis of the mean value of image contrast threshold for different image series numbers of the CDMAM 3.4 phantom using CDMAM Analysis 2.3.0 software (NCCPM)

	The mean value of the threshold image contrast for a given number of images ± 2*SE [μm]:								Maximum value –threshold contrast	Minimum value –threshold contrast	Difference (max–min)/ min [%]
number of analyzed images (Sx):	2	4	6	8	12	16	24	32			
Diameter gold disc [mm]											
0.10	0.747 ± 0.223	0.760 ± 0.144	0.769 ± 0.112	0.772 ± 0.093	0.746 ± 0.070	0.735 ± 0.057	0.741 ± 0.044	0.746 ± 0.037	0.772	0.735	5.12
0.13	0.481 ± 0.128	0.488 ± 0.083	0.497 ± 0.066	0.490 ± 0.054	0.483 ± 0.041	0.471 ± 0.033	0.472 ± 0.026	0.478 ± 0.021	0.497	0.471	5.57
0.16	0.349 ± 0.090	0.355 ± 0.060	0.361 ± 0.048	0.354 ± 0.039	0.353 ± 0.031	0.343 ± 0.025	0.343 ± 0.020	0.348 ± 0.017	0.361	0.343	5.40
0.20	0.251 ± 0.064	0.256 ± 0.044	0.261 ± 0.035	0.255 ± 0.029	0.256 ± 0.023	0.250 ± 0.019	0.249 ± 0.015	0.252 ± 0.013	0.261	0.249	4.65
0.25	0.185 ± 0.046	0.190 ± 0.032	0.193 ± 0.026	0.190 ± 0.021	0.191 ± 0.017	0.187 ± 0.014	0.187 ± 0.011	0.188 ± 0.010	0.193	0.185	4.05
0.31	0.141 ± 0.036	0.145 ± 0.025	0.147 ± 0.020	0.145 ± 0.017	0.146 ± 0.013	0.144 ± 0.011	0.144 ± 0.009	0.145 ± 0.008	0.147	0.141	3.89
0.40	0.106 ± 0.029	0.108 ± 0.020	0.109 ± 0.016	0.108 ± 0.013	0.108 ± 0.010	0.108 ± 0.009	0.108 ± 0.007	0.108 ± 0.006	0.109	0.106	2.94
0.50	0.083 ± 0.026	0.084 ± 0.017	0.084 ± 0.014	0.084 ± 0.011	0.084 ± 0.009	0.084 ± 0.008	0.084 ± 0.006	0.084 ± 0.005	0.084	0.083	1.08
0.63	0.067 ± 0.025	0.066 ± 0.016	0.066 ± 0.012	0.065 ± 0.011	0.065 ± 0.008	0.066 ± 0.007	0.066 ± 0.005	0.065 ± 0.004	0.067	0.065	3.09
0.80	0.055 ± 0.026	0.052 ± 0.016	0.052 ± 0.012	0.051 ± 0.010	0.051 ± 0.008	0.052 ± 0.006	0.052 ± 0.005	0.051 ± 0.004	0.055	0.051	7.47
1.00	0.047 ± 0.028	0.043 ± 0.016	0.043 ± 0.012	0.042 ± 0.009	0.041 ± 0.007	0.042 ± 0.006	0.041 ± 0.004	0.041 ± 0.003	0.047	0.041	13.94

S—CDMAM 3.4 phantom designation

x—number of analyzed images

This pattern is consistent across most diameters. For 1.00 mm disks, the threshold image contrast ranged from 0.047 ± 0.028 (2 images) to 0.041 ± 0.003 (32 images), with a maximum difference of 13.94%, the highest among all diameters. In contrast, for 0.50 mm disks, the measured contrast remained remarkably stable (range: 0.083–0.084), with only a 1.08% difference, indicating low variability at this diameter.

Statistical analysis using the Tukey test for the CDMAM 3.4 phantom revealed significant differences between the following groups: S2 vs S6 (*p* < 0.006), S6 vs S16 (*p* < 0.001), S6 vs S24 (*p* < 0.002), S6 vs S32 (*p* < 0.021), S8 vs S16 (*p* < 0.019), and S8 vs S24 (*p* < 0.048). Additional differences identified with the more liberal NIR test included: S2 vs S8 (*p* < 0.004), S4 vs S16 (*p* < 0.011), S4 vs S24 (*p* < 0.008), S6 vs S12 (*p* < 0.011), and S8 vs S32 (*p* < 0.022).

Analyzing the results presented in Table 1, it is not possible to clearly determine the trend of increase or decrease in the values for individual diameters in individual groups of image numbers. Only a decrease in the standard errors is observed with the increase in the number of images analyzed in the group. The largest difference between the absolute values of the results obtained for individual groups of images was recorded for a diameter of 1 mm and the smallest for 0.5 mm. Also, in the case of differences between the limit values, it is not possible to indicate a specific trend depending on the size of the disk diameter.

Table 2 presents the same analysis for the CDMAM 4.0 phantom. Here, a more consistent improvement in precision is observed with increasing numbers of images. For a 0.10 mm disk, the threshold image contrast decreased from 0.843 ± 0.146 (2 images) to 0.799 ± 0.029 (32 images), with a 6.68% difference and a fivefold reduction in standard error. This trend of decreasing contrast and error is seen across nearly all diameters, especially those below 0.5 mm.

**Table 2:** Analysis of the mean value of image contrast threshold for different image series numbers of the CDMAM 4.0 phantom using CDMAM Analysis 2.3.0 software (NCCPM)

	The mean value of the threshold image contrast for a given number of images ± 2*SE [μm]:								Maximum value –threshold contrast	Minimum value –threshold contrast	difference (max–min)/ min [%]
number of analyzed images (Nx):	2	4	6	8	12	16	24	32			
Diameter gold disc [mm]											
0.1	0.843 ± 0.146	0.808 ± 0.094	0.811 ± 0.075	0.807 ± 0.063	0.801 ± 0.050	0.801 ± 0.042	0.791 ± 0.033	0.799 ± 0.029	0.843	0.791	6.68
0.13	0.560 ± 0.086	0.539 ± 0.057	0.539 ± 0.046	0.541 ± 0.04	0.537 ± 0.032	0.537 ± 0.028	0.531 ± 0.022	0.534 ± 0.019	0.560	0.531	5.44
0.15	0.454 ± 0.066	0.437 ± 0.045	0.436 ± 0.037	0.439 ± 0.032	0.435 ± 0.026	0.436 ± 0.022	0.432 ± 0.018	0.433 ± 0.016	0.454	0.432	5.05
0.18	0.351 ± 0.049	0.338 ± 0.033	0.337 ± 0.027	0.340 ± 0.024	0.337 ± 0.019	0.337 ± 0.017	0.335 ± 0.014	0.335 ± 0.012	0.351	0.335	4.81
0.21	0.287 ± 0.039	0.277 ± 0.026	0.275 ± 0.022	0.278 ± 0.019	0.276 ± 0.015	0.276 ± 0.013	0.274 ± 0.011	0.274 ± 0.009	0.287	0.274	4.74
0.25	0.230 ± 0.030	0.221 ± 0.020	0.220 ± 0.016	0.222 ± 0.014	0.220 ± 0.012	0.220 ± 0.010	0.219 ± 0.008	0.219 ± 0.007	0.230	0.219	4.89
0.3	0.184 ± 0.024	0.177 ± 0.016	0.176 ± 0.013	0.177 ± 0.011	0.176 ± 0.009	0.176 ± 0.008	0.175 ± 0.006	0.175 ± 0.005	0.184	0.175	5.15
0.35	0.153 ± 0.020	0.147 ± 0.014	0.146 ± 0.011	0.147 ± 0.009	0.146 ± 0.007	0.146 ± 0.006	0.145 ± 0.005	0.146 ± 0.004	0.153	0.145	5.23
0.42	0.125 ± 0.017	0.121 ± 0.012	0.120 ± 0.009	0.120 ± 0.008	0.119 ± 0.006	0.119 ± 0.005	0.119 ± 0.004	0.119 ± 0.004	0.125	0.119	5.56
0.5	0.104 ± 0.015	0.100 ± 0.010	0.099 ± 0.008	0.099 ± 0.007	0.099 ± 0.005	0.099 ± 0.005	0.098 ± 0.004	0.099 ± 0.003	0.104	0.098	5.61
0.57	0.090 ± 0.013	0.087 ± 0.009	0.086 ± 0.007	0.086 ± 0.006	0.086 ± 0.005	0.086 ± 0.004	0.086 ± 0.003	0.086 ± 0.003	0.090	0.086	5.61
0.66	0.078 ± 0.011	0.075 ± 0.007	0.074 ± 0.006	0.074 ± 0.005	0.074 ± 0.004	0.074 ± 0.004	0.074 ± 0.003	0.074 ± 0.003	0.078	0.074	5.83
0.77	0.067 ± 0.009	0.065 ± 0.006	0.064 ± 0.005	0.064 ± 0.004	0.064 ± 0.004	0.064 ± 0.003	0.064 ± 0.003	0.064 ± 0.002	0.067	0.064	5.48
0.88	0.060 ± 0.008	0.058 ± 0.006	0.057 ± 0.004	0.057 ± 0.004	0.057 ± 0.003	0.057 ± 0.003	0.057 ± 0.002	0.057 ± 0.002	0.060	0.057	5.27
1.0	0.054 ± 0.008	0.052 ± 0.005	0.052 ± 0.004	0.051 ± 0.003	0.051 ± 0.003	0.051 ± 0.002	0.051 ± 0.002	0.052 ± 0.002	0.054	0.051	4.68

N—CDMAM 4.0. phantom designation

x—number of analyzed images

In this phantom, the Tukey test revealed a statistically significant difference between the N2 group and all other groups (*p* < 0.000), confirmed by the NIR test. Additionally, the NIR test indicated significance between N4 vs N24 (*p* < 0.023) and N8 vs N24 (*p* < 0.022). These differences support the conclusion that at least 24 images should be used for stable and low-error measurements.

For larger diameters (above 0.57 mm), the threshold image contrast remains nearly unchanged across all image group sizes, indicating measurement stability regardless of sample size. For instance, for a 1.00 mm disk, the contrast ranged only from 0.054 to 0.051, and the difference between maximum and minimum values was just 4.68 %. Analyzing the results presented in Table 2, with the increase in the size of the group for individual disk diameters, the absolute value of the threshold image contrasts and the standard error decrease. This trend is less observed for diameters above 0.5 mm—but it still exists. The differences between the minimum and maximum values between groups of images for individual disk diameters do not show a systematic change (no change trend). This proves that the size of the disk diameter does not affect the reading in the CDMAM 4.0 phantom.

In Table 2, the measurement precision improves markedly with an increasing number of analyzed images. For a 0.10 mm disk, contrast values decrease from 0.843 ± 0.146 (2 images) to 0.799 ± 0.029 (32 images). The most substantial decrease in standard error occurs between 2 and 12 images. Beyond 24 images, changes are minimal, indicating stability. Coefficient of variation dropped from ~ 17% to ~ 3.6% for the smallest disk diameter, confirming better precision for small structures.

## Discussion

### Clinical implications

From a clinical point of view, the threshold image contrast is an extremely important physical parameter. High image contrast guarantees that all the smallest changes that may be present in the examined breast (microcalcifications) are recorded in the image. It should be remembered that the threshold image contrast improves with the increase in the dose emitted by the X-ray tube during each projection of the mammographic examination. The increase in the dose of ionizing radiation contradicts the basic principles of radiological protection of the patient. Since mammographic examinations are performed mainly in women as part of a screening program, special care should be taken to ensure the level of exposure to ionizing radiation of potentially healthy women. When calibrating mammography devices with a digital image detector, regardless of whether they are CR or DR mammography devices, special attention should be paid to setting the optimal exposure parameters that will provide an appropriately low dose of ionizing radiation or a high threshold image contrast. Such a setting will provide a high-quality image while maintaining the low dose of ionizing radiation that women receive during a mammography examination. It is therefore extremely important that the user of the mammography device ensures periodic measurement of the threshold image contrast on their device and measurement of the dose in a correct manner and in accordance with available scientific knowledge.

Underestimating the threshold image contrast due to analyzing too few images may result in falsely assuming the imaging system is performing within acceptable limits. In practical terms, this can lead to failure in detecting subtle but clinically important features such as microcalcifications, especially at early stages of breast cancer. Furthermore, inadequate image sampling may reduce the sensitivity of quality control protocols, masking gradual degradation in detector performance or exposure system calibration. This, in turn, could affect compliance with accreditation standards (e.g., EUREF or national QA programs) and potentially delay necessary maintenance or corrective actions. Therefore, ensuring an adequate number of images during CDMAM testing is not only a technical consideration but also a factor directly influencing diagnostic confidence and patient safety.

According to the EUREF recommendations from 2006, to obtain the correct value of the threshold image contrast, it was required to analyze at least 6 images in the "for processing" format. These recommendations changed in 2013, where it was recommended to perform the analysis of at least 16 images in the "for processing" format. The image analysis software provided by the image manufacturer requires the analysis of at least 8 images of the CDMAM phantom. It should be added here that between subsequent exposures the phantom should be moved so that the disks are not located over the same pixels, which ensures the extraction of information from the image about artifacts not related to the image detector. It can therefore be seen that the number of analyzed images has a key impact on the measurement result. It is important to use images in the analysis in the "for processing" format, which are characterized by a linear relationship between dose and pixel value. The use of images in the "for processing" format ensures the repeatability and reliability of the obtained analysis results. In the images in the "for processing" format, CAD (Computed Aided Detection/Diagnosis) software has not applied filters to improve the visibility of anatomical structures and bring their appearance and contrast closer to analog images. The use of such filters does not allow for maintaining the repeatability of the analysis of images obtained from different types of mammograms. Unfortunately, there is no research in the available scientific literature on the number of CDMAM phantom images analyzed for which the obtained threshold image contrast result will be the best.

Maintaining low threshold image contrast, especially for small diameter disks, ensures visibility of microcalcifications—key in early breast cancer detection. For CDMAM 4.0, the use of 24 images improved precision of measurements for 0.1 mm disks by approximately 1.2%, reducing uncertainty from ~ 3.6% to ~ 2.4%. Though this may appear minor, even small improvements in contrast detection can affect sensitivity in borderline cases.

### Technical considerations

In principle, with the increase in the number of analyzed images, the threshold image contrast value should be obtained, which is precisely determined, and its standard error is smaller. This situation is generally observed in Tables 1 and 2. In the case where the number of images analyzed had no effect on the obtained result, all values of the threshold image contrast should have the same value. The results obtained show that this is not the case.

To determine the optimal number of analyzed images, at which the result is the most reliable, two important parameters must be considered. The first is the obtained result of the threshold image contrast. According to the principles of statistics, it must take the values as small as possible with a simultaneous low standard error. The second is the number of analyzed images. This number must be low enough to perform the test using the CDMAM phantom in an acceptable time. Several or a dozen measurements can be performed at an acceptable time, while several hundred measurements are impossible to perform during routine operation of the device.

The low value of the threshold image contrast is particularly important in the context of controlling the exposure automation system. In accordance with the EUREF recommendations, the value of the threshold image contrast for a disk diameter of 0.1 mm is considered in the test for compensation for changes in the phantom thickness and the value of the high voltage. Therefore, an appropriately low and thus reliably determined value of the threshold image contrast for a diameter of 0.1 mm significantly affects the verification of the correct operation of the exposure automation, which in current mammographs is crucial for obtaining images of the highest diagnostic value.

In the CDMAM 3.4 phantom, it is not possible to clearly determine which group of results is characterized by the lowest or highest values. From the point of view of statistics, the highest values of the threshold image contrast should be obtained for the group in which only two images were analyzed—in this case, the highest values are obtained in the group in which 6 images were analyzed. It is not possible to indicate the group in which the threshold image contrast values were the lowest. This situation is very clearly visible in the statistical analysis performed using Tukey and NIR tests. Due to the large diversity of the threshold image contrast results in individual groups, many different groups of results are recorded that differ statistically from each other, which is clearly visible in the statistics performed. This means that it is not possible to clearly state how many images of the CDMAM 3.4 phantom should be analyzed to obtain the optimal result of the threshold image contrast. One can only venture to say that from the point of view of the threshold image contrast value for a 0.1 mm diameter disk, it is best to use 16 images for analysis—if we use this value to evaluate the exposure automation system. In the opinion of the authors of this paper, the EUREF recommendations regarding the number of images analyzed should be followed for the CDMAM 3.4 phantom. This suggestion does not stem from the present data, which showed inconsistent trends across groups and diameters, but rather reflects alignment with the EUREF 2013 guidelines. Given the observed variability in results, we recommend continuing with the existing standard of 16 images, particularly for 0.1 mm disks used in automatic exposure control tests, until more robust evidence allows for an updated recommendation.

The situation is completely different when analyzing groups of images obtained using the CDMAM 4.0 phantom. Statistical analysis showed that there is only a statistically significant difference between the group and the other groups and N4 vs. N24 and N8 vs. N24. Analyzing the values in the individual groups, it is clearly visible that the lowest values were shown in the group in which 24 images were analyzed and the highest in the group in which 2 images were analyzed. To obtain optimal results, it is recommended to analyze at least 24 images of the CDMAM 4.0 phantom based on the measurements performed. With such analysis, we obtain results characterized by the smallest values with the smallest standard error (the results are precisely determined). EUREF recommends taking at least 16 images every 6 months. If it takes about 1 min to obtain one image in a DR mammogram, the total test time increases by about 10 min at most. An increase in the number of images analyzed by 8 will not significantly extend the time of performing this test, especially since we perform it every 6 months. An increase in the number of analyzed images increases the precision of the measurement by approx. 1.2% in the range of small disk diameters, which is particularly important in the context of the exposure automation control test.

It is also worth noting that in the case of both phantoms, the differences in absolute values between the individual groups become blurred with the increase in the diameter of the analyzed disks. This is consistent with theoretical predictions. Although this tendency is better observed for CDMAM 4.0 phantom than for CDMAM 3.4, in the CDMAM 4.0 phantom, the threshold image contrast practically does not change for disks above 0.57 mm.

In the above analysis, software from one manufacturer was used, allowing for the simultaneous analysis of two types of phantoms. However, it should be remembered that the method of reading images is the responsibility of cdcom.exe files/programs, which are a fundamental part of every software for reading CDMAM images. It is responsible for, among other things, detecting disks on the phantom, smoothing the detection matrix with a Gaussian filter, and fitting a polynomial to the thickness-diameter relationship. There are different versions of cdcom.exe, which have been improved over the years and for phantom versions. It is cdcom.exe that is largely responsible for the result of the analysis with the CDMAM phantom and thus the analyses presented in this work. From a practical standpoint, implementing a 24-image protocol for CDMAM 4.0 phantom analysis appears feasible in most clinical settings, particularly those using DR systems with automated acquisition. While doubling the number of images from 12 to 24 increases test time, the gain in measurement precision is especially notable for small diameters. Facilities with resource constraints may adopt a tiered approach—performing 24 images biannually during full QA cycles and fewer images during interim checks.

### Limitations

One of the limitations of this study was the inability to determine an optimal number of images for CDMAM 3.4. Although statistical testing showed differences between several image groups, the mean contrast values fluctuated nonlinearly, and no stable minimum could be identified across all diameters. This may result from higher inherent variability of the phantom structure, lower object count per diameter, or limitations in phantom design. Additionally, the relatively smaller number of total repetitions (10 per group) may have influenced the statistical robustness. As a result, the study supports adherence to EUREF recommendations (≥ 16 images) for CDMAM 3.4 but does not provide a new, evidence-based recommendation for clinical users.

Of course, the results of this work may turn out to be different in the case of a significant increase in the number of repetitions in individual groups. However, in the authors’ opinion, the trends described for individual versions of CDMAM phantoms will not change significantly. Another certain limitation of this work is the fact that the images were obtained from only one mammogram (i.e. only with one exposure parameter).

The generalizability of these findings is limited by the use of a single mammography system (Hologic DR) and a fixed exposure parameter set. Results may differ with other vendors or technologies (e.g., CR systems), due to variations in detector characteristics, image processing algorithms, and exposure control methods.

It would be necessary to check in later studies how the described trends are presented on mammograms from other manufacturers or on CR (Computed Radiography) mammograms. At the same time, it should be remembered that a properly functioning digital mammograph has no problems with obtaining an image that meets the threshold image contrast requirements specified in the EUREF Guidelines. This can be seen in the presented analysis. Regardless of the number of images analyzed, the results in a properly functioning mammograph will be within the tolerance range. Of course, if the device functions at the border of acceptance (this can happen especially in the case of CR systems), the number of analyzed images is very important. The differences between the minimum and maximum values between groups, regardless of the phantom version, are at the level of about 5% (Table 1* and *Table 2). The threshold image contrast must meet the requirements for disks with a diameter of 0.1, 0.25, 0.5, and 1 mm. If even one diameter does not fit within the specified limit, the device must be repaired accordingly. The absolute value of the threshold image contrast for a disk diameter of 0.1 mm is only used in the automatic exposure test. Therefore, every care must be taken to ensure that the threshold image contrast is reliably determined for this disk diameter.

## Conclusions

From the studies conducted, it is not possible to clearly determine the most advantageous number of images required for analysis for the CDMAM 3.4 phantom. In this case, it is necessary to rely on the manufacturer’s recommendations, which are related to the software requirements. For the CDMAM 4.0 phantom, to obtain reliable results, it is recommended to perform 24 images for analysis. However, it should be noted that performing 8 images does not significantly worsen the obtained results; it only doubles the error of the obtained result, which is not a premise for accepting the test. Special attention should be paid when determining the threshold image contrast for a disk diameter of 0.1 mm because this parameter is used during the control of exposure automation. These results should be interpreted in the context of a single-system study design. Future investigations across multiple systems and vendors, including CR technology, are warranted to assess the broader applicability of the findings.

## Data Availability

All measurement results performed in this work will be made available by the corresponding author upon request. During the preparation of this work, the authors did not use AI technology.
